# Northern Ohio Dental Association

**Published:** 1867-08

**Authors:** 


### NORTHERN OHIO DENTAL ASSOCIATION,

Held its annual meeting at Cleveland, on the 7th and 8th of May. The election of officers for the ensuing year resulted in the choice for President of Dr. B. F. Robinson; Vice-President, Dr. C. H. Harroun; Recording Secretary, W. P. Horton; Corresponding Secretary, Dr. C. R. Butler; Treasurer, Dr. Chas. Buffett.

Delegates to the American Dental Association, Drs. F. S. Whitslar, Dr. Templeton, C. H. Harroun, C. C. Carroll, B. F. Robinson, Chas. Buffett and J. E. Robinson.

Essayists were appointed upon various subjects.

This meeting was well attended, and much interest was manifested in the general subject.

W. P. Horton, *Secretary.*

